# Pathogenic *REST* variant causing Jones syndrome and a review of the literature

**DOI:** 10.1038/s41431-022-01258-9

**Published:** 2022-12-13

**Authors:** Elisa Rahikkala, Johanna Julku, Sari Koskinen, Tommi Keski-Filppula, Stephanie Weissgraeber, Aida M. Bertoli-Avella, Sanna Häkli, Minna Kraatari-Tiri

**Affiliations:** 1 PEDEGO Research Unit, University of Oulu, Oulu, Finland; 2 Department of Clinical Genetics and Medical Research Center, Oulu University Hospital, Oulu, Finland; 3 Department of Oral and Maxillofacial Diseases, Oulu University Hospital, Oulu, Finland; 4 Department of Medical Reporting and Genomics, Centogene GmbH, Rostock, Germany; 5 Department of Otorhinolaryngology and Phoniatrics, Oulu University Hospital, Oulu, Finland

**Keywords:** Disease genetics, Genetic predisposition to disease

## Abstract

Jones syndrome is a rare dominantly inherited syndrome characterized by gingival fibromatosis and progressive sensorineural hearing loss becoming symptomatic in the second decade of life. Here, we report a father and his two daughters presenting with a typical Jones syndrome (OMIM %135550) phenotype. Exome sequencing identified a repressor element 1-silencing transcription factor (*REST*, OMIM *600571) (NM_005612.5) c.2670_2673del p.(Glu891Profs*6) heterozygous variant segregating with Jones syndrome in the family. We review the clinical data from all previously published patients with Jones syndrome and previously published patients with pathogenic *REST* variants associated with gingival fibromatosis or sensorineural hearing loss. This study suggests that pathogenic *REST* variants cause Jones syndrome.

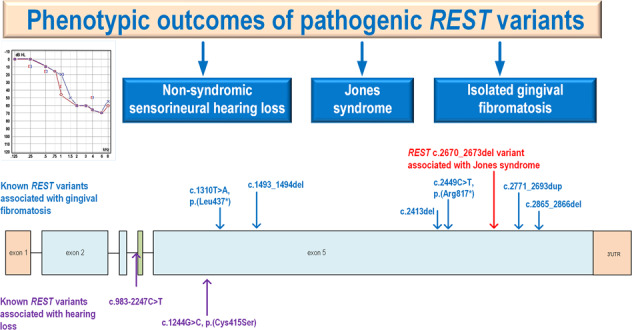

## Introduction

In 1977, Jones et al. described five generations of a family with autosomal dominantly inherited gingival fibromatosis (GF) and progressive sensorineural hearing loss (SNHL) becoming symptomatic late in the second decade of life [1]. The affected individuals had hypertrophied, pink, stippled, and nodular gingiva, predominantly in the anterior part of the mouth and predominant premaxillae. Clinical and audiometric examinations revealed progressive downward-sloping SNHL. The syndrome was named Jones syndrome (OMIM %135550), and several families with Jones syndrome were later described in the medical literature [2–5]. However, the underlying gene causing Jones syndrome remains elusive.

Pathogenic variants in the repressor element 1-silencing transcription factor (*REST)* gene (OMIM *600571) have previously been associated with dominantly inherited SNHL [6, 7], GF [8–10], or susceptibility to Wilms tumor [11]. Here, we describe a Finnish family with a typical Jones syndrome phenotype. Exome sequencing identified a pathogenic *REST* variant segregating with the phenotype. To our knowledge, this is the first report of the association between a pathogenic *REST* variant and Jones syndrome.

## Subjects and methods

### Molecular genetics

Whole-exome sequencing (WES) was performed for Patients 1–3 in Centogene (Rostock, Germany). Targeted Sanger sequencing was used for segregation analysis of the identified *REST* variant. Details of sequencing and bioinformatics are included in the supplement.

### Clinical data

**Fig. 1:**
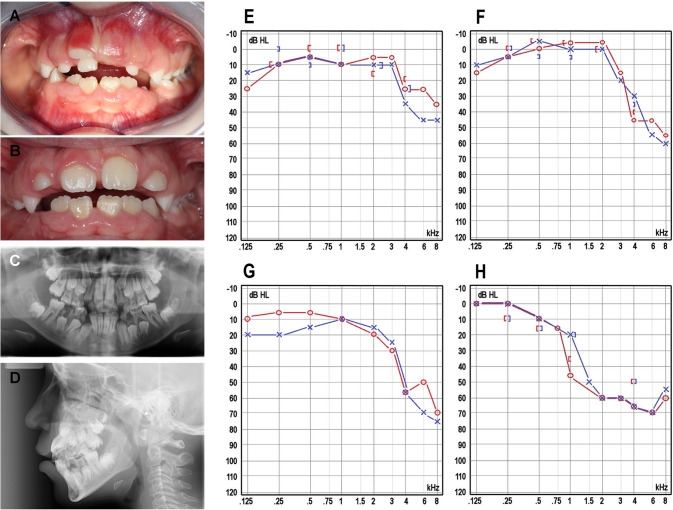
Clinical characteristics of affected individuals. **A**. Patient 1 at age 10 with marked gingival fibromatosis, which prolonged the retention of primary teeth and delayed permanent tooth eruption. **B**. Gingiva of Patient 1 after surgical treatment. **C**. A pantomogram of Patient 1 showing that all the unerupted teeth were present with normal root development. **D**. A cephalogram of Patient 1 showing a convex profile with an open bite tendency. Audiograms of Patient 1 at age 11 (**E**.) and Patient 2 at age 14 (**F**) show mild bilateral SNHL affecting the high frequencies. Audiograms of Patient 3 at age 5 (**G**) and age 42 (**H**) show the progression of SNHL from initially affecting only the high frequencies to gradually affecting the middle frequencies as well.

*Patient 1* is an 11-year-old female (Supplementary Fig. 1, II-5). After birth, transient evoked otoacoustic emissions were not obtained, but an automated auditory brainstem response was obtained bilaterally. Her growth and development were normal. At age 10, she was referred for further investigations due to GF prolonging the retention of primary teeth and delaying permanent tooth eruption (Fig. 1A). Dental panoramic tomography (DPT) showed that all the unerupted teeth were present and had normal root development (Fig. 1C), and lateral cephalometric radiograph showed a convex profile with an open bite tendency (Fig. 1D). Extractions of over-retained primary teeth and the removal of excessive gingiva covering non-erupted permanent teeth were performed to ensure the eruption of the teeth (Fig. 1B). An audiogram revealed mild bilateral SNHL affecting the high frequencies (Fig. 1E). She does not need hearing aids and has mild hypertrichosis and hypertelorism.

*Patient 2*, an older sister of Patient 1, is a 14-year-old female. Her growth and development were normal. At the age of five years, mild bilateral SNHL affecting the high frequencies was identified (Fig. 1F). No remarkable progression has been noted, and she does not need hearing aids. She has GF, which has delayed permanent tooth eruption. The DPTs showed normal root development, with delayed eruption because of primary retention. Cephalometric analysis showed sagittal jaw discrepancy with neutral facial growth. She also has mild hypertrichosis.

*Patient 3*, the father of Patients 1–2, is a 42-year-old male, whose parents and siblings do not have SNHL or GF. At the age of one year, bilateral inguinal hernias were corrected surgically. At age four, right-sided Legg-Perthes-Calve disease was treated with osteotomy. His bone age was delayed. He has mild hypertrichosis. At age five, he was diagnosed with bilateral SNHL affecting predominantly high frequencies (Fig. 1G), and his hearing loss has slowly progressed. His latest audiograms at age 40 revealed SNHL affecting the middle and high frequencies (Fig. 1H). He is using hearing aids. At age ten, he was referred to a dentist due to markedly delayed (2.5 years) permanent tooth eruption. At age 14, he was referred to an orthodontist due to a convex profile with a retrognathic mandible, and labially inclined upper incisors with an excessive overjet and open bite. There was also generalized GF preventing the eruption of several permanent teeth. He was treated with orthodontics as a teenager, and a gingivectomy was performed at age 20. At follow-up, GF had not recurred.

## Results

### Whole-exome sequencing

WES of Patients 1–3 identified a heterozygous *REST* (NM_005612.5) c.2670_2673del p.(Glu891Profs*6) (GRCh38 NC_000004.12:g.56931528_56931531del) variant (Supplementary Fig. 2). Sanger sequencing of the unaffected siblings confirmed the segregation (Supplementary Fig. 1). The variant is not present in the Genome Aggregation Database (v.2.1.1). It is classified as pathogenic (PVS1, PM2, PP1) according to the ACMG criteria [12].

#### Review of the literature

The clinical and genetic details of the *REST-*related phenotypes are summarized in Table 1 and pathogenic *REST* variants reported in the literature are shown in Supplementary Fig. 3.

**Table 1:** Known pathogenic variants of the *REST* gene causing hearing loss (HL) or gingival fibromatosis (GF) and the clinical findings of cases reported.

Study	*REST* variant (NM_005612.5)	Phenotype	Origin	Age (years)	Sex	Age of onset for GF (years)	Age of onset/age of first audiological examination^a^ for HL (years)
This study	c.2670_2673del; p.(Glu891Profs*6)	Jones syndrome	Finland	11	F	10	11
This study	c.2670_2673del; p.(Glu891Profs*6)	Jones syndrome	Finland	14	F	13	4
This study	c.2670_2673del; p.(Glu891Profs*6)	Jones syndrome	Finland	42	M	10	5
Machado et al. [10]	c.1493_1494del, p.(Glu498Glyfs*17)	GF	Brazil	53	M	ND	No HL
Machado et al. [10]	c.1493_1494del, p.(Glu498Glyfs*17)	GF	Brazil	57	M	ND	No HL
Machado et al. [10]	c.1493_1494del, p.(Glu498Glyfs*17)	GF	Brazil	22	F	ND	No HL
Machado et al. [10]	c.1493_1494del, p.(Glu498Glyfs*17)	GF	Brazil	25	F	ND	No HL
Chen et al. [9]	c.2449C>T; p.(Arg817*)	GF	Taiwan	46	M	ND	No HL
Chen et al. [9]	c.2771_2793dup; p.(Glu932Lysfs*3)	GF	Taiwan	15	F	ND	No HL
Bayram et al. [8]	c.2865_2866delAA; p.(Asn958Serfs*9)	GF	Turkey	17	M	7-8	No HL
Bayram et al. [8]	c.2865_2866delAA; p.(Asn958Serfs*9)	GF	Turkey	48	M	ND	No HL
Bayram et al. [8]	c.2865_2866delAA; p.(Asn958Serfs*9)	GF	Turkey	13	M	7–8	No HL
Bayram et al. [8]	c.1310T>A; p.(Leu437*)	GF	Turkey	9	M	3–4	No HL
Bayram et al. [8]	c.1310T>A; p.(Leu437*)	GF	Turkey	38	F	3–4	No HL
Bayram et al. [8]	c.1310T>A; p.(Leu437*)	GF	Turkey	14	F	3–4	No HL
Bayram et al. [8]	c.1310T>A; p.(Leu437*)	GF	Turkey	16	M	3–4	No HL
Bayram et al. [8]	c.1310T>A; p.(Leu437*)	GF	Turkey	32	M	3–4	No HL
Bayram et al. [8]	c.2413delC; p.(Leu805Phefs*38)	GF	USA	9	F	3–4	No HL
Manyisa et al. [6]	c.1244G>C; p.(Cys415Ser)	BSNHL	South Africa	12	M	No GF	3
Manyisa et al. [6]	c.1244G>C; p.(Cys415Ser)	BSNHL	South Africa	37	F	No GF	27
Peters et al. [13]	c.983-2247C>T	BSNHL	USA	NA	F	No GF	ND
Peters et al. [13]	c.983-2247C>T	BSNHL	USA	69	M	No GF	69^a^
Peters et al. [13]	c.983-2247C>T	BSNHL	USA	47	M	No GF	33^a^
Peters et al. [13]	c.983-2247C>T	BSNHL	USA	35	F	No GF	35^a^
Peters et al. [13]	c.983-2247C>T	BSNHL	USA	65	M	No GF	65^a^
Peters et al. [13]	c.983-2247C>T	BSNHL	USA	64	M	No GF	64^a^
Peters et al. [13]	c.983-2247C>T	BSNHL	USA	41	M	No GF	28^a^
Peters et al. [13]	c.983-2247C>T	BSNHL	USA	57	F	No GF	57^a^
Peters et al. [13]	c.983-2247C>T	BSNHL	USA	42	F	No GF	42^a^
Peters et al. [13]	c.983-2247C>T	BSNHL	USA	41	F	No GF	32^a^
Peters et al. [13]	c.983-2247C>T	BSNHL	USA	39	F	No GF	27^a^
Peters et al. [13]	c.983–2247C>T	BSNHL	USA	28	M	No GF	28^a^

*BSNHL* bilateral sensorineural hearing loss, *F* female, *M* male, *ND* not determined.

^a^If the age of onset was not available, we have reported the age of first audiological examination showing hearing loss.

#### Previously published patients with Jones syndrome

Twelve patients with Jones syndrome have been described previously (Supplementary Table 1) [1–5]. All had GF, which was often severe, and five required surgical treatment. Histology of the extracted gingiva showed thickened epithelium, elongated rete ridges, inflammation, and dense fibrous connective tissue stroma. Ten had SNHL, and audiogram results often moderate to severe SNHL affecting mainly higher frequencies. The average age of onset for SNHL was 14 years (10–20 years), and for GF 9 years (4–20 years). Other findings included hirsutism, hypermobility of the distal phalanges, hypertrophic scar, undescended testis, and intellectual disability.

#### Pathogenic REST variants causing gingival fibromatosis

An original report described nine individuals from three families with GF caused by three different pathogenic *REST* final-exon-truncating variants [8]. Chen et al. reported two patients with GF caused by two novel truncating pathogenic *REST* variants [9]. In vitro, reported gene assays demonstrated that these truncated RESTs had a partial or complete loss of repressor activity and that truncated RESTs impaired the repressive ability of the wild-type REST, suggesting a dominant negative effect [9]. Recently, Machado et al. reported a novel truncating *REST* variant segregating with mild GF in a Brazilian family [10].

#### Pathogenic REST variants causing sensorineural hearing loss

Peters et al. mapped a dominantly inherited postlingual progressive SNHL to the *REST*-containing *DFNA27* locus in chromosome 4 [13]. Sequencing of all annotated exons in the *DFNA27* locus failed to identify potentially pathogenic variants, but sequencing of conserved intronic regions of *REST* revealed a heterozygous intronic variant segregating with a hearing loss phenotype in the family [7]. This intronic *REST* variant (NM_005612.5) c.983-2247C>T resulted in the prevention of alternative splicing of the REST mRNA, which was essential for the regulation of *REST* in the inner ear [7]. Manyisa et al. reported on a South African family presenting with progressive non-syndromic SNHL caused by the *REST* c.1244G>C, p.(Cys415Ser) missense variant [6]. The affected individuals in these two families showed either a flat-type audiogram affecting all frequencies or a downward-sloping audiogram affecting mostly the high frequencies.

## Discussion

This brief report describes a father and his two daughters presenting with GF and progressive SNHL, also known as Jones syndrome. WES identified a small deletion in the last exon of *REST*, segregating with the phenotype.

*REST* encodes a transcriptional regulator expressed in most non-neuronal cells and its expression is minimal in maturing hair cells and most neurons [7]. It contains a DNA- binding domain that can bind to a specific 21 base pair consensus sequence (RE-1) in the promoter region of its target genes to repress the transcription of the target genes [14]. REST acts as a master negative regulator of neurogenesis, a regulator of osteoblast differentiation, and a key repressor of gene expression in hypoxia [15].

Previously, pathogenic *REST* variants were associated with progressive dominantly inherited SNHL in two families [6, 7]. The affected individuals in these two families had either a flat-type audiogram affecting all the frequencies or downward-sloping audiograms, similar to Patients 1–3 described in this report. REST is also involved in the organization of the auditory receptor cell stereocilium [15]. REST expression was decreased in hair cells and spiral ganglion neurons in age-related hearing loss in mice. REST has a protective role in age-related hearing loss, and its deficiency upregulates p53 and induces cochlear cell apoptosis, which then leads to SNHL [14].

The TGF-β/Smad signaling cascade and overproduction of the extracellular matrix (ECM), especially collagen type I, have been proposed to account for GF [16, 17]. REST is expressed in the epithelium and lamina propria of normal and fibrotic gingiva [9], and it controls gingival homeostasis by supressing profibrotic genes and activating proteolytic genes [9]. It is speculated that loss-of-function *REST* variants cause increased production but reduced turnover of the ECM leading to GF [10].

Previously, a deep intronic *REST* c.983-2247C>T variant has been demonstrated to cause dysregulation of alternative splicing and deafness and a gain-of-function has been suggested as a disease mechanism [7]. This variant is not present in any of the three affected individuals from this study, and therefore it can be excluded as causing SNHL in our patients. Recent studies in mice seem to contradict the gain-of-function hypothesis as deletion of *REST* in the cochlea resulted in hearing impairment and induced apoptosis of spiral ganglion neurons and hair cells [14], suggesting that loss of *REST* function may increase the susceptibility to SNHL. *REST* c.2670_2673del is in the last exon and leads to protein truncation probably escaping nonsense-mediated decay suggesting that susceptibility to SNHL and gingival hypertrophy may be caused by a loss-of-function mechanism. Also, a dominant negative mechanism has been suggested as a disease mechanism in patients with pathogenic *REST* variants [9]. However, we cannot exclude the possibility that SNHL in our family would be caused by a yet unidentified pathogenic variant.

It is possible that patients reported in ref. [6–10] actually had Jones syndrome as SNHL and gingival hypertrophy may be mild or even non-penetrant [1] and not routinely sought after in patients with gingival or audiological phenotypes. While further studies are required to confirm the association between *REST* and Jones syndrome, based on our study, we recommend detailed oral and hearing examinations of patients with potentially pathogenic heterozygous *REST* variants. As SNHL in young patients can be relatively mild, predominantly affecting high frequencies and progressing slowly, it may be undiagnosed without proper audiological testing.

Jones syndrome is a rare, possibly underdiagnosed, slowly progressive disease. Early diagnosis and follow-up allow timely treatment of GF and hearing loss, decreasing the severity of the disease and improving patients’ quality of life.

## Supplementary information

Supplemental File

## Data Availability

The reported variant was submitted to the LOVD database hosted at Leiden University Medical Center, the Netherlands (DB-ID REST_000030).
