# Supplementary material for: Pathogenic REST variant causing Jones syndrome and a review of the literature
Source: Eur J Hum Genet. 2022 Dec 13;31(4):469–73. doi: 10.1038/s41431-022-01258-9 (PMC10133349; doi:10.1038/s41431-022-01258-9)
Supplement: Supplementary file 1 — Supplemental File [file 41431_2022_1258_MOESM1_ESM.docx]

**SUPPLEMENTARY MATERIAL**

**Pathogenic *REST* variant causing Jones syndrome and a review of the literature**

Elisa Rahikkala^1,2^, Johanna Julku^3^, Sari Koskinen^3^, Tommi Keski-Filppula^1,2^, Stephanie Weissgraeber^4^, Aida M. Bertoli-Avella^4^, Sanna Häkli^1,5^, Minna Kraatari-Tiri^1,2^

^1^ PEDEGO Research Unit, University of Oulu, Oulu, Finland

^2^ Department of Clinical Genetics and Medical Research Center, Oulu University Hospital, Oulu, Finland

^3^ Department of Oral and Maxillofacial Diseases, Oulu University Hospital, Oulu, Finland

^4^ Department of Medical Reporting and Genomics, Centogene GmbH, Rostock, Germany

^5^ Department of Otorhinolaryngology and Phoniatrics, Oulu University Hospital, Oulu, Finland

**Methods**

*Exome sequencing*

Exome sequencing was performed as described previously (1). In brief, genomic DNA was enzymatically fragmented, and target regions are enriched using DNA capture probes. These regions include approximately 41 Mb of the human coding exome (targeting > 98% of the coding RefSeq from the human genome build GRCh37/hg19), as well as the mitochondrial genome. The generated library is sequenced on an Illumina platform to obtain at least 20x coverage depth for > 98% of the targeted bases. An in-house bioinformatics pipeline, including read alignment to GRCh37/hg19 genome assembly and revised Cambridge Reference Sequence (rCRS) of the Human Mitochondrial DNA (NC_012920), variant calling, annotation, and comprehensive variant filtering is applied. All variants with minor allele frequency (MAF) of less than 1% in gnomAD database, and disease-causing variants reported in HGMD®, in ClinVar or in CentoMD® (2) were evaluated. The investigation for relevant variants is focused on coding exons and flanking +/-10 intronic nucleotides of genes with clear gene-phenotype evidence (based on OMIM® information). Variants were classified categorized into five classes (pathogenic, likely pathogenic, VUS, likely benign, and benign) along ACMG guidelines (3).

Primers were designed according to the reference sequence of *REST* NM_005612.5. and containing the variant c.2670_2673del p.(Glu891Profs*6) (primers available upon request). Sanger sequencing was performed in both forward and reverse direction on a 3730xl sequencer (Thermo Fisher Scientific, Waltham, MA).

**Results**


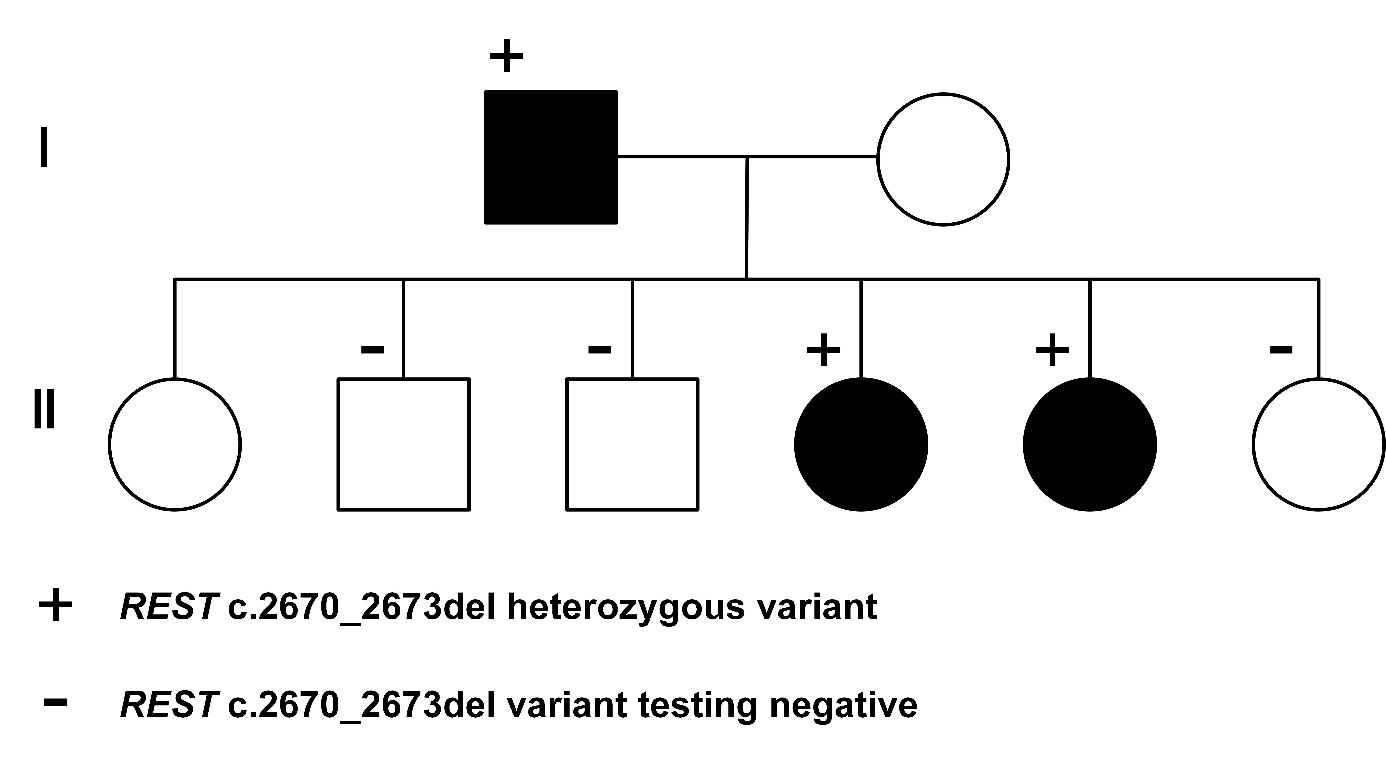


**Supplementary Figure 1.** Pedigree of the family. *REST* (NM_005612.5) heterozygous c.2670_2673del p.(Glu891Profs*6) variant segregated dominantly with the Jones syndrome phenotype.


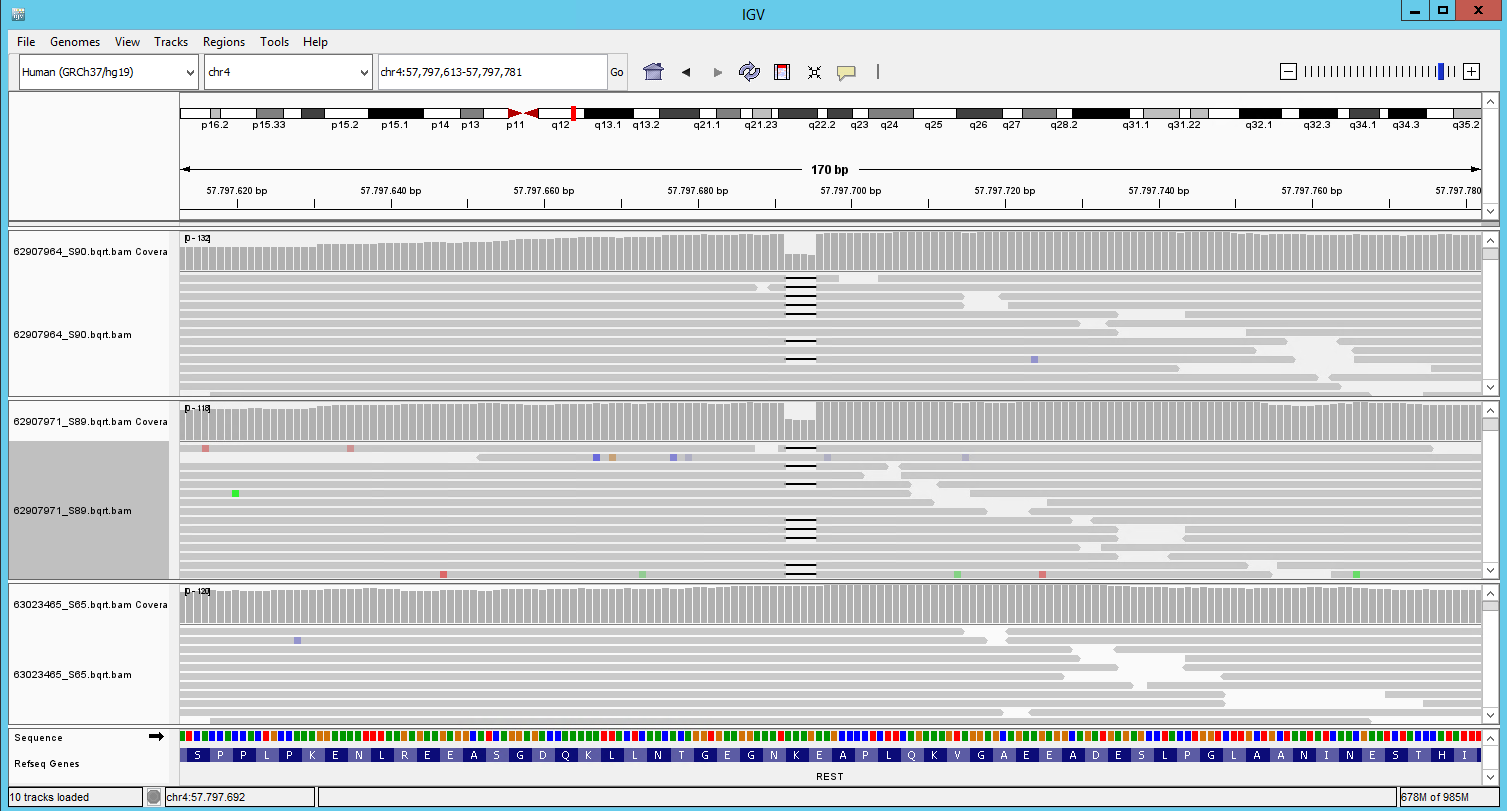


**Supplementary Figure 2.** Integrative genomics viewer (IGV) image from the index patient (above), the affected father (in the middle), and an unaffected healthy control (below) individual showing the region on the *REST* with the small heterozygous c.2670_2673del deletion.

*
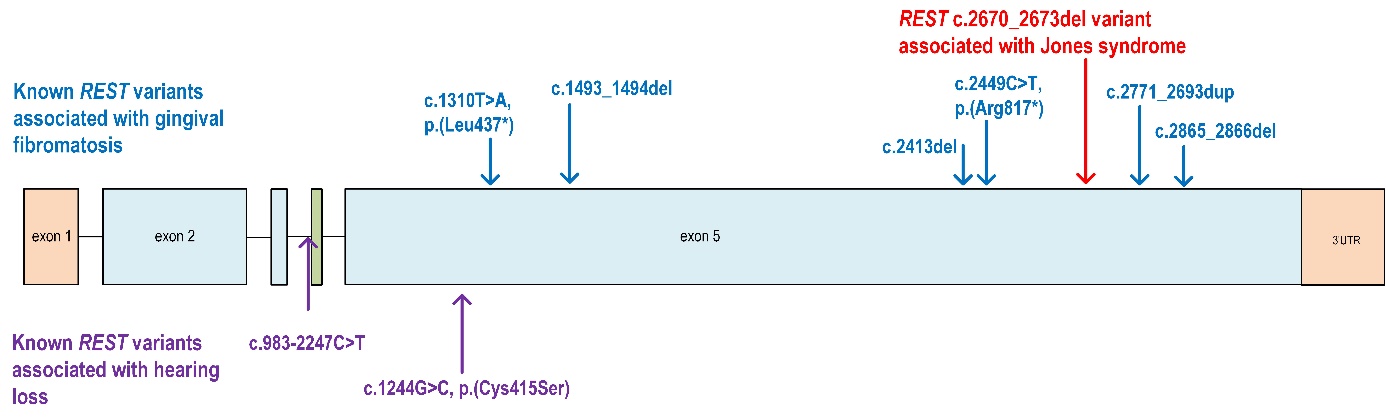
*

**Supplementary Figure 3.** Known pathogenic variants in the *REST* gene associated with gingival fibromatosis or hearing loss. Exons are to scale, whereas the introns and UTRs (in pink) are not to scale. Exon 4 (in green) is an alternative exon, and regulated alternative splicing of this exon is important for hearing (4). *REST* c.2670_2673del variant described in this study is shown above the gene (in red). Known pathogenic *REST* variants associated with gingival fibromatosis are shown above the gene (in blue), and known pathogenic *REST* variants associated with hearing loss are shown below the gene (in purple). Isoform reference is *REST*: NM_005612.5.

**Supplementary Table 1.** Clinical characteristics of previously published patients with Jones syndrome.

| Study | Origin | Age (y) | Sex | Oral findings | Histology | Treatment | Hearing phenotype | Treatment | Age of onset for GF (y) | Age of onset for SNHL (y) | Family history | Other findings |
| --- | --- | --- | --- | --- | --- | --- | --- | --- | --- | --- | --- | --- |
| Jones et al. 1977 (5) | USA | 11 | M | Gingival fibromatosis, most prominent anteriorly, gingival tissue extends over the teeth. Prominent premaxilla. Disorientation of unerupted teeth, deviation from normal eruption angle. | Edema, moderate vascularity of lamina propria, thickening of superficial epithelium, increased size of rete ridges, increased fibroblast epithelial metaplasia | NA | Bilateral significant SNHL above 2,000 Hz | NA | NA | NA | Yes | Broad alveolar ridges, large and protruded pinnae |
| Jones et al. (1977) (5) | USA | 43 | F | Gingival fibromatosis, most prominent anteriorly. Hypertrophic, pink, stippled, and nodular gingiva. Prominent premaxilla. | NA | NA | SNHL of 60 dB at 6,000 Hz and 45 dB in lower frequencies | No, reads lips | NA | 10-20 | Yes | No |
| Jones et al. (1977) (5) | USA | 60 | M | Gingival fibromatosis | NA | Complete dental extraction and gingivectomy | SNHL of 60 dB at frequencies above 1,500 Hz, SNHL of 30 dB below 1,500 Hz | NA | NA | <40 | Yes | No |
| Jones et al. (1977) (5) | USA | 20 | F | Gingival fibromatosis, prominent premaxilla | NA | No | No SNHL | No | NA | No | Yes | No |
| Jones et al. (1977) (5) | USA | 38 | F | Gingival fibromatosis | NA | Gingivectomy, extraction of maxillary and mandibular incisors | Bilateral SNHL of 30–40 dB below 1,500 Hz |  |  |  |  |  |
| Jones et al. (1977) (5) | USA | NA (>20) | F | Gingival fibromatosis | NA | NA | No SNHL | No | NA | No | Yes | No |
| Hartsfield et al. (1985) (6) | USA | 10 | F | Generalized gingival fibromatosis, covering most of the anatomic crowns of teeth. Delayed eruption of permanent incisors and first molars. | NA | NA | Mild sloping SNHL (20– 40 dB) between 4,000–8,000 Hz on left, borderline acuity on right | NA | NA | NA | Yes | Delayed eruption of teeth (13 months), slight posterior angulation of ears |
| Hartsfield et al. (1985) (6) | USA | 33 | M | Marked gingival fibromatosis. Mild to moderate bone loss around molars and incisors | NA | NA | Moderate sloping SNHL (30–70 dB) between 4,000–8,000 Hz in right ear, between 1,000–8,000 Hz in left | NA | NA | NA | Yes | Sparse hair on head, no hair on arms. Hypermobility of the distal phalanges. |
| Hartsfield et al. (1985) (6) | USA | NA | M | Gingival fibromatosis covering major portion of dental crowns, midline torus palatinus. | NA | NA | Bilateral, sloping, moderate (30–70 dB) SNHL between 2,000–8,000 Hz | NA | NA | NA | Yes | Obesity, hirsutism at birth, thick body hair, hypertrophic scar |
| Kasaboglu et al. (2004) (7) | Turkey | 42 | M | Severe, generalized gingival fibromatosis on palate and vestibular side of upper molars. Dense, fibrous consistency with marked grooving on the hard palate. Cystic lesion in right maxillary molar region. | Increased hypocellular, hypovascular collagenous mass under thickened epithelium, elongated rete ridges, inflammation | Excision of fibromatous gingiva, enucleation of cyst, no recurrence after surgery. | Moderate bilateral SNHL after 2,000 Hz | No | 20 | NA | Yes | Undescended testis bilaterally, infertility |
| Gita et al. (2014) (8) | India | 14 | M | Generalized gingival fibromatosis. Firm, nodular, and fibrotic gingiva. Heavy melanin pigmentation, lower anterior teeth barely visible. No bone loss. | Parakeratinized stratified squamous epithelium with underlying dense fibrous connective tissue stroma, fibromatous epithelium, prominent, elongated rete ridges | Gingivectomy, mild recurrence after surgery. | Severe to profound SNHL in right, moderately severe to severe SNHL in left | High occlusion computer generated hearing aids | 13 | 13 | No | No |
| Da et al. (2016) (9) | India | 15 | M | Severe generalized gingival fibromatosis in both dental arches. Firm, nodular, fibrous, and erythematous gingiva. Plaque and calculus covering more than half of the crown portion. Generalized horizontal bone loss. | Parakeratinized stratified squamous acanthotic epithelium, long thin rete ridges, connective tissue showed dense bundles of collagen fibers, infiltration of chronic inflammatory cells, multinucleated giant cells, areas of neovascularization | External bevel gingivectomy, no recurrence after surgery. | Progressive SNHL | No | 15 | NA | No | Intellectual disability |

Abbreviations: M, male; F, female; y, years; SNHL, sensorineural hearing loss; GF, gingival fibromatosis

**References**

1. Trujillano D, Bertoli-Avella AM, Kumar Kandaswamy K, Weiss ME, Köster J, Marais A, ym. Clinical exome sequencing: results from 2819 samples reflecting 1000 families. Eur J Hum Genet. 2017; https://doi.org/10.1038/ejhg.2016.146.

2. Trujillano D, Oprea GE, Schmitz Y, Bertoli-Avella AM, Abou Jamra R, Rolfs A. A comprehensive global genotype-phenotype database for rare diseases. Mol Genet Genomic Med. 2017; https://doi.org/10.1002/mgg3.262.

3. Richards S, Aziz N, Bale S, Bick D, Das S, Gastier-Foster J, et al. Standards and guidelines for the interpretation of sequence variants: a joint consensus recommendation of the American College of Medical Genetics and Genomics and the Association for Molecular Pathology. Genet Med. 2015; https://doi.org/10.1038/gim.2015.30.

4. Nakano Y, Kelly MC, Rehman AU, Boger ET, Morell RJ, Kelley MW, et al. Defects in the Alternative Splicing-Dependent Regulation of REST Cause Deafness. Cell. 2018; https://doi.org/10.1016/j.cell.2018.06.004.

5. Jones G, Wilroy RS, McHaney V. Familial gingival fibromatosis associated with progressive deafness in five generations of a family. Birth Defects Orig Artic Ser. 1977; 13(3B):195–201

6. Hartsfield JK, Bixler D, Hazen RH. Gingival fibromatosis with sensorineural hearing loss: an autosomal dominant trait. Am J Med Genet. 1985; https://doi.org/10.1002/ajmg.1320220323.

7. Kasaboğlu O, Tümer C, Balci S. Hereditary gingival fibromatosis and sensorineural hearing loss in a 42-year-old man with Jones syndrome. Genet Couns. 2004;15(2):213–8.

8. Gita B, Chandrasekaran S, Manoharan P, Dembla G. Idiopathic gingival fibromatosis associated with progressive hearing loss: A nonfamilial variant of Jones syndrome. Contemp Clin Dent. 2014; https://doi.org/10.4103/0976-237X.132387.

9. Da R, Singh S, Gupta I, Gopal S. Gingival Enlargement in a Case of Variant Jones Syndrome: a Case Report. J Dent (Shiraz). 2016; 17(1):62–6.
